# Autoimmune diseases share a common genetic architecture involving the JAK-STAT pathway

**DOI:** 10.17179/excli2024-7684

**Published:** 2024-08-27

**Authors:** Iraj Saadat, Mostafa Saadat

**Affiliations:** 1 Department of Biology, School of Science, Shiraz University, Shiraz 71467-13565, Iran

## ⁯⁯⁯⁯⁯

The function of the immune system is to protect the host from infection. Discrimination between two sets of self and non-self antigens is critical and very important to the function of the immune system. Autoimmune diseases result from an abnormal response by the adaptive immune system that mistakenly attacks the body's own cells, causing damage to tissues and organs. There are more than 80 autoimmune diseases, and some recent scientific evidence suggests that there may be as many as 100 autoimmune diseases (Li et al., 2024[8]). They are costly to treat and currently rely on non-specific and universal immunosuppression that only provides symptomatic relief without addressing the underlying causes. The most common diseases commonly classified as autoimmune include rheumatoid arthritis (RA), systemic lupus erythematosus (SLE), multiple sclerosis (MS), ulcerative colitis (UC), and Crohn's disease (CD). 

Nineteen of the most common autoimmune diseases are estimated to affect approximately one in ten in the general population (Conrad et al., 2023[3]). Familial co-association studies have shown that type 1 diabetes and Hashimoto's thyroiditis often cluster in individuals and families, suggesting a common origin in both genetic and environmental factors (Skov et al., 2022[15]).

Familial and twin studies have shown a higher concordance rate of autoimmune diseases in monozygotic twins than in dizygotic or sibling pairs, indicating the role of genetic susceptibility. In the group of autoimmune diseases, the heritability ranges from 0.008 to 1, with a median of about 0.60 (Selmi et al., 2012[14]). It is possible that autoimmune diseases share a common genetic basis. This means that the molecular mechanisms that eventually lead to autoimmune diseases are triggered by genetic changes in specific genes. Due to the lack of research in this area, the present study was conducted.

Polymorphic loci have been associated with the risk of RA, SLE, MS, UC, and CD were extracted from the GWAS database (https://www.ebi.ac.uk/gwas) on December 2023 using the keywords "rheumatoid arthritis", "systemic lupus erythematosus", "multiple sclerosis", "ulcerative colitis", and "Crohn's disease". 

A total of 155, 72, 68, 66, and 71 studies were included in the database for RA, SLE, MS, UC, and CD diseases, respectively. A total of 3277, 1534, 798, 801, and 1092 significant associations were initially extracted for RA, SLE, MS, UC, and CD diseases, respectively. Only protein-coding genes were included in the present analysis. Any gene with at least one significant polymorphism association with susceptibility to RA, SLE, MS, UC, and CD was included in the analysis. A total of 174, 293, 201, 110, and 143 polymorphic protein-coding loci were identified whose genetic polymorphisms were associated with the risk of RA, SLE, MS, UC, and CD, respectively. 

The data sets were then further analyzed using Venn diagrams to find common loci between RA, SLE, MS, UC, and CD (Supplementary information, Figure 1). For all diseases, the majority (45-70 %) of the polymorphic loci were unique. Note that 109, 206, 130, 49, and 71 loci were associated only with RA, SLE, MS, UC, and CD, respectively. The remaining genes were common to varying degrees among the diseases studied. Some were common between two, three, or four, and even all five diseases (all diseases studied). The distribution of polymorphic genes among the studied diseases is summarized in the Supplementary information, Table 1.

There were 153 polymorphic genes that were associated with at least two of the study diseases. The present data showed that 117, 24, 10, and 2 genes were shared between two, three, four, and five diseases, respectively. Finally, *STAT4*, and *TYK2* genes were common to all diseases, and ten loci *ANKRD55*, *BACH2*, *CD226*, *IL2RA*, *JAZF1*, *PLCL1*, *PTPN2*, *RASGRP1*, *STAT3*, and *UBE2L3* were common to four of the diseases. Given the potentially important role of the above 12 genes in the pathogenesis of RA, SLE, MS, UC and CD, enrichment analysis was performed on these genes.

We used the Enrichr online server ([link:http://maayanlab.cloud/Enrichr*http://maayanlab.cloud/Enrichr]) to perform Gene Ontology (GO), Reactome, and Kyoto Encyclopedia of Genes and Genomes (KEGG) analysis. In the present study, Reactome 2022 and KEGG 2021 databases were used to retrieve pathways for pathway enrichment analysis. Gene ontology (GO) 2023 enrichment analysis was performed, including molecular functions, cellular components, and biological processes as described previously (Saadat, 2023[13]). The results of KEGG and Reactome analysis are shown in the Supplementary information, Table 2. KEGG analysis showed that the Janus kinase/signal transduction and activator of transcription (JAK-STAT) pathway was the top predicted pathway (adjusted p-value = 1.3e-6). Genes encoding STAT4, TYK2, IL2RA, PTPN2, and STAT3 were involved in the JAK-STAT pathway. This means that 50 % of the genes shared between autoimmune diseases belong to the JAK-STAT pathway. Reactome analysis also showed that the interlukin-23, interlukin-35, interlukin-20 family, interlukin-2 family pathways were the top predicted pathways (adjusted p-value < 1.0e-5). Both analyses indicated that the majority of genes analyzed were involved in immune system function. 

The present study showed that 12 genes are common to at least 4 of the 5 diseases studied. Enrichment analysis revealed that 50 % of the shared genes (STAT4, TYK2, IL2RA, PTPN2, and STAT3) among autoimmune diseases belong to the JAK-STAT pathway. It can be concluded that the pathogenesis of SLE, RA, MS, UC, and CD share a common molecular basis, and the basis is the activation of the JAK-STAT pathway.

The JAK-STAT signaling pathway is involved in the control of a variety of biological processes, such as the inflammatory response (Hu et al., 2023[7]). There is evidence that it has been used for over 500 million years for intracellular signaling in response to cytokines and is therefore highly conserved throughout biological evolution, indicating its importance (Agaisse and Perrimon, 2004[1]). Several lines of evidence suggest that genes involved in the JAK-STAT pathway are involved in autoimmune diseases, including a decrease in autoantibody titers is associated with inhibition of the JAK-STAT pathway (Wang et al., 2010[16]), *STAT4*-deficient mice are resistant to models of autoimmune disease (Finnegan et al., 2002[5]), and susceptibility to autoimmune disease is associated with polymorphisms in the *TYK2* and *STAT4* genes (Remmers et al., 2007[12]; Liang et al., 2012[9]; Ebrahimiyan et al., 2019[4]; Pellenz et al., 2021[11]). In autoimmune diseases, multiple circulating cytokines activate different combinations of JAKs/STATs to transduce intracellular signals and subsequently alter the cell fate in target tissues and induce end-organ damage (Banerjee et al., 2017[2]; Hu et al., 2023[7]). 

Today, many researchers believe that targeting the JAK-STAT pathway is a milestone in the treatment of autoimmune diseases. JAK inhibitors have shown potential in the treatment of SLE. Among these, TYK2 inhibition is emerging as a promising therapeutic strategy (Nikolopoulos and Parodis, 2023[10]; Yuan et al., 2023[17]). In models of arthritis, specific targeting of STAT4 with antisense oligonucleotides can ameliorate disease, suggesting that STAT4 may be a useful therapeutic target (Hildner et al., 2007[6]). On the other hand, the association of polymorphisms of genes involved in the JAK-STAT pathway with the risk of several autoimmune diseases (Liang et al., 2012[9]; Ebrahimiyan et al., 2019[4]; Pellenz et al., 2021[11]) has provided a very interesting research area for the development of a laboratory diagnostic kit to identify the high-risk individuals for developing autoimmune diseases. 

## Conflict of interest

The authors declare no conflict of interest.

## Supplementary Material

Supplementary information
