# Supplementary material for: Autoimmune diseases share a common genetic architecture involving the JAK-STAT pathway
Source: EXCLI J. 2024 Aug 27;23:1027–9. doi: 10.17179/excli2024-7684 (PMC11483264; doi:10.17179/excli2024-7684)
Supplement: Supplementary information [file EXCLI-23-1027-s-001.pdf]

EXCLI Journal 2024;23:1027-1029 – ISSN 1611-2156
Received: January 24, 2024, accepted: April 26, 2024, published: August 27, 2024

Supplementary information to:

Letter to the editor:

AUTOIMMUNE DISEASES SHARE A COMMON GENETIC ARCHITECTURE INVOLVING THE JAK-STAT PATHWAY

Iraj Saadat*, Mostafa Saadat

Department of Biology, School of Science, Shiraz University, Shiraz 71467-13565, Iran

* **Corresponding author:** Iraj Saadat, Department of Biology, School of Science, Shiraz University, Shiraz 71467-13565, Iran; Fax: +98-71-32280926;
E-mail: isaadat@shirazu.ac.ir

https://dx.doi.org/10.17179/excli2024-7684

This is an Open Access article distributed under the terms of the Creative Commons Attribution License (http://creativecommons.org/licenses/by/4.0/).

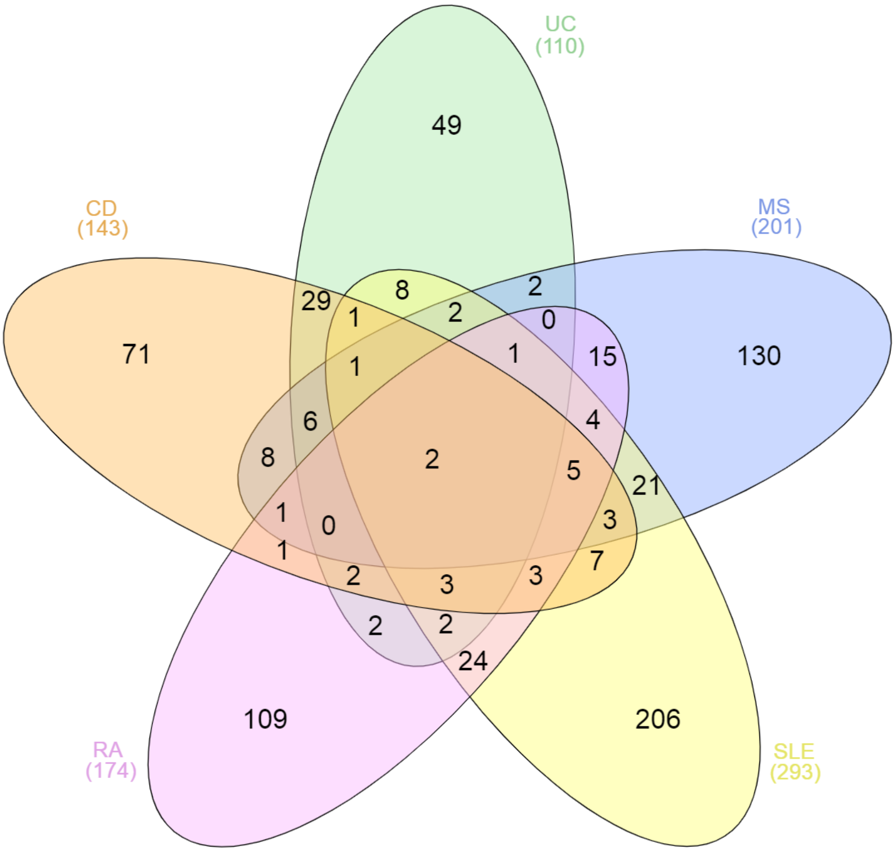

Supplementary Figure 1: Venn diagram showing the distribution of polymorphic loci in rheumatoid arthritis (RA), systemic lupus erythematosus (SLE), multiple sclerosis (MS), ulcerative colitis (UC), and Crohn's disease (CD)

EXCLI Journal 2024;23:1027-1029 – ISSN 1611-2156
Received: January 24, 2024, accepted: April 26, 2024, published: August 27, 2024

Supplementary Table 1: The distribution of polymorphic genes among the studied diseases rheumatoid arthritis (RA), systemic lupus erythematosus (SLE), multiple sclerosis (MS), ulcerative colitis (UC), and Crohn's disease (CD)

Diseases	Number	Genes
CD MS RA SLE UC	2	TYK2 STAT4
MS RA SLE UC	1	CD226
CD MS RA SLE	5	IL2RA RASGRP1 ANKRD55 BACH2 JAZF1
CD RA SLE UC	3	PLCL1 PTPN2 UBE2L3
CD MS SLE UC	1	STAT3
MS RA SLE	4	PXT1 COG6 CD40 NAB1
RA SLE UC	2	PRKCB HLA-DRB9
CD RA SLE	3	PTPN22 KSR1 ATXN2
CD MS RA	1	IFNGR2
CD RA UC	2	DAP BLTP1
MS SLE UC	2	IL7R HLA-DQA1
CD MS SLE	3	LPP SKAP2 RMI2
CD SLE UC	1	ZNF831
CD MS UC	6	INAVA CD6 RGS14 ADCY3 NDFIP1 PUS10
RA SLE	24	RTKN2 IRAK1 RAD51B TET3 TNFSF4 MECP2 IL12RB2 TNPO3 ARID3A IRF5 ARID5B IL6R TRAF1 TNIP1 MTF1 FAM193A ATG5 WDFY4 SPRED2 ILF3 MACIR TNFAIP3 BLK AFF3
MS RA	15	GATA3 GPC5 ZFP36L1 IQGAP1 PLCL2 TTC34 OS9 CASP8 FCRL3 ELMO1 GFI1 ARHGEF3 MMEL1 BATF CSF2RB
RA UC	2	PRDM16 GSDMB
CD RA	1	NFATC1
MS SLE	21	PHLDB1 AFF1 SLC44A2 GRB2 DOCK10 TRAF3 IKZF3 TIMMDC1 MYNN TERT EVI5 AHI1 LEF1 CLEC16A ATXN1 NCOA2 ETV7 GALC MANBA LBH IQCB1
SLE UC	8	IL19 IL10 TNXB HLA-DRA ETS1 CFB ZFP90 PTPRC
CD SLE	7	BANK1 SBNO2 MAGI3 JAK2 ELF1 FCHSD2 IL18RAP
MS UC	2	CDH3 TET2
CD MS	8	RUNX3 ZBTB38 PHACTR2 SP140 CDC37 ZMIZ1 FOXP1 DNMT3A
CD UC	29	HORMAD2 INTS11 FCGR2A ERAP2 GPR65 BSN SMAD3 DENND1B OSMR TNFSF15 GATD3 GPR35 GLYAT LITAF IL23R CARD9 CELSR3 MAP3K8 BRD2 PARK7 RORC SPATA48 EEF1AKMT2 MST1 ZGPAT NOTCH1 CDKAL1 EPHB4 IL27
RA	109	TPD52 PSTPIP1 NTRK1 ANAPC4 C11orf21 RMND5A TMEM235 PDE2A VPS37C CCR6 CDK5RAP2 CXCL13 FCGR2B PHACTR3 ANXA3 CUL5 RIN3 CCN4 AIFM2 NEDD4 PPIL4 DPP4 IKZF1 BOLL FLT3 AIRE CYRIB SWAP70 PLD4 STAG1 DNASE1L3 RBFOX1 SMC1B DENND1A ZPBP2 TMEM151B TCERG1 SLC9A9 ZNF774 CDC42EP3 RAB5B ATP6V1G3 TEC SH2B3 CD247 ARAP1 ANTXR2 TNFRSF11A RUNX1 SMTNL2 PADI2 ZNF366 AHR MSI1 CASTOR1 SH3PXD2A HAPLN4 NUAK1 TXNDC11 ZNF679 MAPK14 CDK6 REL ACOXL C5 NECAB2 TMEM179 NFKBIE PRKCQ DLGAP1 RCAN1 GMCL2 TBC1D8 RGS10 PRKCH PHF19 TPCN2 SFTPD FAM107A KIF5A GNG4 ARL15 FCGR3A PREX1 ZNF689 SIMALR RNF19A SDF2L1 GCH1 CPA4 KMT2B ARHGAP26 ICOSLG TRHDE CCDC201 MANEAL TTC33 ALPK1 TMEM187 RAVER1 SYT16 IL2RB SUOX SYNGR1 KCP SPC24 UBASH3A TPRA1 DRD1
SLE	206	PPARD ANKS1A TMEM86B ITGAM CLNK C4B MUC22 PHRF1 ARHGAP31 SLC17A1 ARMC3 SRCAP SYT1 CYCS ERI1 DPF3 TCP11 STN1 ANKRD16 IKZF2 CHD9 DAB2

EXCLI Journal 2024;23:1027-1029 – ISSN 1611-2156
Received: January 24, 2024, accepted: April 26, 2024, published: August 27, 2024

		IRF1 SFTA2 TMEM39A PLD2 BLTP3A FAP NSF GTF2H1 PRAG1 PRSS54 TAOK3 SMO RPS20 ETV3 LRRK1 CPNE5 CD80 PDCD5 OR4A15 HIBCH C10orf67 SMYD3 PSORS1C1 PLAT FBN2 PUSL1 IKBKB SSBP4 PAPOLG CTLA4 UBAC2 CABP1 KDM4C ST8SIA4 TRAPPC11 C3 TRIM31 AP5B1 CDKN1B SLC15A4 IQCJ-SCHIP1 TMC2 SLC12A1 PUS7 FAM98B FNIP1 RPS6KA4 SPPL3 MGAT5 CARMIL1 C1QTNF6 R3HCC1 DDX6 IPCEF1 FCGR2C PLEKHM1 GABRR2 G6PD PRPS2 LDB2 STIM1 NRG3 SLC17A3 NTNG2 GPR19 RGL1 MYO5C BACE2 CFH ARHGAP4 ABHD8 KDM4B C2 TNIP2 RASGRP3 CYP4F8 CENPU TMEM116 DARS1 ARHGAP15 CDHR5 HLA-DQB1 CAMK1D SLC9A4 SLC17A2 NADSYN1 TASL ITGAX SBK1 FDX2 FCRL5 ZNF804B NMNAT2 NOTCH4 SORCS2 LRIT2 MSRA NEURL4 STK19 TNFRSF13B XKR6 GLS ICAM3 MTG1 CCDC113 C1QTNF12 ANXA6 ICA1 CSK ZSCAN23 OAS1 CDH23 ZNF76 NT5C2 LYST HLA-DPA1 SIGLEC5 GTF2I SPIN3 LAMC2 LRRC56 TENT4A IRF7 FBXL20 OLFML3 KIT HLA-A LCP1 DSE SCGN RABGAP1L EIF4H IFIH1 FDFT1 SLC29A3 CSNK2B SEZ6L2 ANKRD27 NCF1 HLA-DQB2 INPP5B MICA DRAM1 TSPAN33 PPCDC OVOL1 DDO GTF2IRD1 NOTCH2 USP18 POM121C KLF12 CER-CAM LAT2 SCAMP5 LST1 FLI1 DGKQ CEPT1 BBIP1 SCN10A HIP1 AHNAK2 SLC6A16 MFHAS1 ACOX2 ELP3 AGXT2 UBE2B KEAP1 ADAM15 HLA-V CTNNA3 PXK TEX51 DOT1L STAT1 PDE4A TP63 GATA4 PRDM1 CREBL2 RERE ACAP1
MS	130	TNIP3 ZHX3 NCOA5 ZNF767P ALDH1L1 SIDT1 MALT1 ERG YWHAG SLC15A2 IFITM3 NCKAP5 RORA CR1L RPS6KA5 SH3BP2 MAPK1 TXK SAMD12 MTHFR CSGALNACT2 PTPN11 NLRC5 RREB1 NCOR2 TTC28 MINK1 POU2AF1 TRIM2 DDAH1 SMARCA4 BCL2 MYO5B FAM76B IFI30 EXOC2 TNFAIP8 JADE2 EGFL6 RNASEL PLEKHG5 HTR3A MAPK10 PRR5L FAM241A HACE1 GTDC1 MERTK PDZRN4 LZTFL1 CHST12 PLXNC1 CENPO CD58 CD48 TOX2 SLAMF7 PDE4B NIHCOLE ZC3HAV1 DIPK1A VMP1 MYBPC3 CBLB LCN6 VANGL2 VTI1A SETD1A ASAP1 AKAP13 MPV17L2 TGFBR3 SLC2A4RG MPHOSPH9 PHKG1 JAK1 UNK FAM167A TNFRSF1A SCAMP2 SGSM2 ZNHIT3 PHGDH SLC9A8 L3MBTL3 RGCC TLL1 SAE1 SLC30A7 PRXL2B AGBL2 DKKL1 LRRIQ4 SLC9B1 PRICKLE1 GNG2 CD69 CSMD1 EPS15L1 PVT1 TRIM14 PITPNM2 ETV6 VAV2 MARCHF1 TSFM TBX6 BRINP1 TNFSF14 WWOX ESPN ADAMTSL1 ZBTB46 CD86 FCRL1 KIF1B CYP24A1 NR1D1 CRTAP TEF LIMK2 RGS1 ALPK2 MCTP1 UCK2 RPS6KB1 FPR1 EXOC6 SPEF2 CXCR5
UC	49	ERGIC1 DPH5 ADCY7 CPXM2 GRAMD1B CACNA2D1 CYTH1 LNC-LBCS LSP1 PLCG2 LAMB1 MAML2 GRID2IP IL17REL RTF1 APEH CCNY HDAC7 CCDC26 CD28 OTUD3 CAMK2A NR5A2 CCHCR1 SEMA6D CIITA DUSP29 BZW2 HDAC9 MROH3P COG2 SATB2 TNRC18 CHP1 CIT SLC39A11 NFKBIZ CCL20 NFKB1 DUSP16 DAPK2 SFMBT1 NXPE1 TOM1 POLR1HASP GNA12 PTGIR SLIT1 ADAD1
CD	71	ADAM30 TUBD1 FIBP IGLV3-21 PLAU RAP1A MUC19 RNF220 C17orf67 VSTM2B IMPG2 ITLN1 PTK2B CD244 SLC22A23 CDH13 BTNL12P C3orf84 FAM171B RFT1 GAL3ST2 NELL1 IRGM GALNTL6 SERPINB6 THADA CPEB4 LRRK2 LACC1 TEX41 GCKR CAVIN1 CNTNAP2 IRF6 PLA2R1 LTBR C1orf141 RIT1 MLN UBE3D ACO2 NRAD1 TRPM3 SCAMP3 DTNB INKA2 ANK2 FERMT1 PLA2G4A CHD5 SBSPON TMEM258 SLAMF8 CDC42SE2 RSPO3 NOD2 FUT2 PER3 DOCK7 SNX20 ATG16L1 HGFAC BTBD8 HUNK TSPAN14 CEP43 C16orf96 CCND3 CLN3 CREM CUL1

EXCLI Journal 2024;23:1027-1029 – ISSN 1611-2156
Received: January 24, 2024, accepted: April 26, 2024, published: August 27, 2024

Supplementary Table 2: Results of Reactome 2022, EKGG, and Gene Ontology enrichment analysis of 12 key polymorphic loci shared by at least four autoimmune diseases: rheumatoid arthritis (RA), systemic lupus erythematosus (SLE), multiple sclerosis (MS), ulcerative colitis (UC), or Crohn's disease (CD)

**Name**	**Genes**	**Adjusted p-value**	**Odds Ratio**	**Combined score**
**Reactome 2022**				
Interleukin-23 Signaling R-HSA-9020933	STAT3, STAT4, TYD2	0.0000015	1110.1	20088.89
Interleukin-35 Signaling R-HSA-8984722	STAT3, STAT4, TYD2	0.0000020	739.96	12678.88
Interleukin-20 Family Signaling R-HSA-8854691	STAT3, STAT4, TYD2	0.0000163	289.35	4244.60
Interleukin-2 Family Signaling R-HSA-451927	IL2RA, STAT3, STAT4	0.0000619	162.17	2115.93
Immune System R-HSA-168256	STAT4, TYK2, CD226, IL2RA, PTPN2, RASGRP1, STAT3, UBE2L3	0.0000622	18.66	239.21
**EKGG 2021**				
JAK-STAT signaling pathway	STAT4, TYK2, IL2RA, PTPN2, STAT3	0.0000012	90.22	1580.05
Th1 and Th2 cell differentiation	STAT4, TYK2, IL2RA	0.0005228	74.53	805.97
Th17 cell differentiation	STAT3, TYK2, IL2RA	0.0005481	63.73	660.36
**GO Biological process 2023**				
Receptor Signaling Pathway via STAT (GO:0097696)	STAT3, STST4, TYK2	0.0001631	237.62	3356.07
Receptor Signaling Pathway via JAK-STAT (GO:0007259)	STAT3, STST4, TYK2	0.0001631	201.57	2753.93
Positive Regulation of Type II Interferon Production (GO:0032729)	CD226, TYK2, RASGRP1	0.0003874	120.81	1474.62
Cellular Response to Interleukin-2 (GO:0071352)	STAT3, IL2RA	0.0003874	799.32	9496.99
Interleukin-2-Mediated Signaling Pathway (GO:0038110)	STAT3, IL2RA	0.0003874	799.32	9496.99
**GO Cellular component 2023**				
Nucleus (GO:0005634)	STAT4, TYK2, PTPN2, STAT3, BACH2, JAZF1, UBE2L3	0.03062	4.85	23.61
Intracellular Membrane-Bounded Organelle (GO:0043231)	STAT4, TYK2, CD226, PTPN2, STAT3, UBE2L3, BACH2	0.03451	4.01	16.30
